# Multicontrast Pocket Colposcopy Cervical Cancer Diagnostic Algorithm for Referral Populations

**DOI:** 10.34133/2022/9823184

**Published:** 2022-08-25

**Authors:** Erica Skerrett, Zichen Miao, Mercy N. Asiedu, Megan Richards, Brian Crouch, Guillermo Sapiro, Qiang Qiu, Nirmala Ramanujam

**Affiliations:** ^1^Department of Biomedical Engineering, Duke University, Durham, NC, USA; ^2^Department of Electrical and Computer Engineering, Purdue University, West Lafayette, IN, USA; ^3^Department of Computer Engineering, Massachusetts Institute of Technology, Cambridge, MA, USA; ^4^Department of Electrical and Computer Engineering, Department of Biomedical Engineering, Department of Computer Science, Department of Mathematics, Duke University, Durham, NC, USA

## Abstract

*Objective and Impact Statement*. We use deep learning models to classify cervix images—collected with a low-cost, portable Pocket colposcope—with biopsy-confirmed high-grade precancer and cancer. We boost classification performance on a screened-positive population by using a class-balanced loss and incorporating green-light colposcopy image pairs, which come at no additional cost to the provider. *Introduction*. Because the majority of the 300,000 annual deaths due to cervical cancer occur in countries with low- or middle-Human Development Indices, an automated classification algorithm could overcome limitations caused by the low prevalence of trained professionals and diagnostic variability in provider visual interpretations. *Methods*. Our dataset consists of cervical images (n=1,760) from 880 patient visits. After optimizing the network architecture and incorporating a weighted loss function, we explore two methods of incorporating green light image pairs into the network to boost the classification performance and sensitivity of our model on a test set. *Results*. We achieve an area under the receiver-operator characteristic curve, sensitivity, and specificity of 0.87, 75%, and 88%, respectively. The addition of the class-balanced loss and green light cervical contrast to a Resnet-18 backbone results in a 2.5 times improvement in sensitivity. *Conclusion*. Our methodology, which has already been tested on a prescreened population, can boost classification performance and, in the future, be coupled with Pap smear or HPV triaging, thereby broadening access to early detection of precursor lesions before they advance to cancer.

## 1. Introduction

### 1.1. Background

Cervical cancer results in over 300,000 deaths each year, 88% of which occur in countries with low- or middle-Human Development Indices (HDI) [1]. Cervical cancer can be prevented with the human papillomavirus (HPV) vaccine and periodic screening and treatment of cervical precancer, as precancerous lesions on the cervix develop into cancer 5-20 years after initial infection with a carcinogenic strain of HPV [2]. In the United States, cervical cancer prevention typically occurs through a three-tiered process. Persons with atypical Papanicolaou (Pap) smears or high-risk HPV subtypes are followed up with colposcopy-guided biopsy; if histopathology results indicate a high-grade precancerous lesion or cancer, the patient is referred for treatment [3]. However, low- and middle-HDI countries that bear the brunt of cervical cancer cases tend to have low rates of screening coverage (<20%), limited access to pathology labs, and low rates of patient follow up (roughly 50%) [4, 5]. Therefore, to avert cervical cancer deaths for those who do not have access to and/or are not eligible for the vaccine, the World Health Organization (WHO) recommends adoption of simplified screening technologies coupled directly with treatment [6].

Visual inspection with acetic acid (VIA) has historically been the most accessible screening option available in low- and middle-income countries (LMICs); however, it remains a poor triage test. VIA has high variability depending on provider experience, patient age, and size of the lesion, and it has diminished sensitivity due to lack of magnification [7–9]. Because there is no capability to capture images with VIA, there is no mechanism or opportunity for quality monitoring [10, 11]. Another method, molecular HPV testing, has been shown to reduce the incidence and mortality from cervical cancer when coupled directly with outpatient treatment for patients with HPV-positive results [8]. Patients with negative HPV results have less than a 1% chance of developing a high-grade precancerous lesion, and the slow progression of the HPV viral infection into cervical cancer suggests that patients would require only one to two screens over their lifetime [12]. However, the effectiveness of an HPV screen-and-treat strategy comes at the cost of overtreatment given that less than half of HPV-positive patients have treatable disease [7]. To reduce overtreatment, many countries have adopted resource-stratified guidelines, recommending that HPV screening be followed by a triage step, such as VIA or colposcopy with biopsy (where available) to confirm the presence of a treatable lesion.

To address limitations with the variability of VIA, our group has developed a low-cost, portable, point-of-care colposcope, called the Pocket colposcope, to replace VIA with high-quality colposcopy, which is the established standard-of-care for triage in high-resource settings [13]. As described in detail by Mueller et al., the Pocket is an FDA-cleared handheld device that is inserted through the speculum and rests on it several centimeters in front of the cervix [14]. Whereas traditional colposcopes weigh 175 pounds, require stable electricity, and cost roughly $20,000, the Pocket colposcope is only 0.25 lb, is powered by a smartphone or tablet, and obtains comparable images at 1/10^th^ the cost. Further, the Pocket colposcope has white light illumination to capture images of the cervix stained with either acetic acid or Lugol’s iodine, and green light imaging capability to accentuate vascular features (features that are observed in a routine colposcopy examination.) Therefore, the Pocket colposcope can overcome the limitations of VIA in settings where traditional colposcopes are too cumbersome and/or expensive to use, while retaining the image quality of these sophisticated devices. However, the limited availability of expert colposcopists in low-resource settings represents yet another bottleneck [7, 9].

### 1.2. Related Works

In the past decade, there has been a growing interest in the development of automated algorithms, often referred to as computer-aided diagnosis (CAD), to address the variability in visual interpretation of colposcopy images [15]. Previous work in image-based cervical cancer classification can be divided into two approaches: (1) the extraction and selection of hand-crafted features from the colposcopy image to be classified by a traditional machine learning model [16–19] and (2) the use of convolutional neural networks (CNNs), which optimize a series of filters to reduce the raw input image into feature maps before the final classification step (Table 1). CNNs for image classification obviate the need for hand crafted features used in traditional machine learning algorithms, which are hard to scale when applied to large heterogenous datasets.

**Table 1:** Related works. Published work exploring cervical cancer classification using colposcopy and cervigram images with CNNs use different databases, but the majority of algorithms are trained and tested on datasets from a single collection site. Common methods of dealing with class-imbalance involve data augmentation or downsampling the negative class.

Title (date)	Methods	Datasets	Results
Automation of Detection of Cervical Cancer Using Convolutional Neural Networks (2018) [20]	*Classification method:* CNN binary classification with acetic acid images *Class imbalance method:* not reported	(1) *Single-clinic private colposcopy data:* 60 negative and 42 positive according to visual inspection	*Training set:* 100% accuracy

An Observational Study of Deep Learning and Automated Evaluation of Cervical Images for Cancer Screening (2019) [21]	*Classification method:* CNN binary classification with acetic acid images *Class imbalance method:* artificial data augmentation	(1) *Guanacaste cervigram data from screening population*: 228 positive according to histological diagnosis and 8,689 negative according to visual inspection	*Validation-test set:* 0.91 AUC

Hybrid Transfer Learning for Classification of Uterine Cervix Images for Cervical Cancer Screening (2019) [22]	*Classification method:* CNN binary classification with acetic acid images *Class imbalance method:* data augmentation to artificially increase size of positive class	(1) *Single-clinic private colposcopy data:* 230 negative and 31 positive according to visual inspection (2) *Guanacaste cervigram data from screening population*: 890 negative images by visual inspection and 523 positive images by histological diagnosis	*Validation set*: 0.85 AUC, 77% sensitivity, and 93% specificity *Testing set:* 0.92 AUC, 89% sensitivity, and 94% specificity

ColpoNet for Automated Cervical Cancer Screening Using Colposcopy Images (2020) [23]	*Classification method:* CNN binary classification with acetic acid images *Class imbalance method:* downsampled negative cases	(1) *Guanacaste cervigram data from screening population*: 400 negative images according to visual inspection and 400 positive images by histological diagnosis	*Testing set:* 81% accuracy

MDFI: Multi-CNN Decision Feature Integration for Diagnosis of Cervical Precancerous Lesions (2020) [24]	*Classification method*: CNN binary classification with acetic acid images *Class imbalance method:* not reported	(1) *Single-clinic private colposcopy data:* 650 positive patient cases and 1059 negative patient cases by histological diagnosis	*Testing set:* 0.84 AUC, 70% sensitivity, and 82% specificity

MSCI: A multistate dataset for colposcopy image classification of cervical cancer screening (2021) [25]	*Classification method*: CNN binary classification with acetic acid time-series, Lugol’s iodine, and green light images *Class imbalance method:* not reported	(1) *Single-clinic private colposcopy data:* 411 normal cases and 268 positive cases by histological diagnosis	*Testing set:* 96% sensitivity and 99% specificity

Multi-state colposcopy image fusion for cervical precancerous lesion diagnosis using BF-CNN (2021) [26]	*Classification method:* CNN binary classification with acetic acid and Lugol’s image pairs *Class imbalance method:* not reported	(1) *Single-clinic private colposcopy data:* 1400 patients: 703 normal cases 797 positive cases by histological diagnosis	*Validation-test set:* 0.909 AUC

A challenge with respect to the development of CNNs is the availability of large datasets. CNNs require hundreds to thousands of images from each class to “learn” the diverse set of features that are not fully represented in small datasets. Given the low prevalence of disease (less than 1 case for every 100 women screened) in a population-based study compared to that in a screen-positive population (10 to 20 cases per 100 women screened), the time required to collect these large datasets can be prohibitive [1, 27–29]. A solution to this problem is to leverage existing databases comprised of Cervigrams and/or standard clinical colposcopy images. However, cervigrams are antiquated, and colposcopes are not accessible in communities with limited resources and infrastructure [4]. As a result, the algorithm developed from existing databases will likely not be translatable without an adequate number of positive cases in the training database from the new device and/or site [30].

### 1.3. Study Overview

Here, we report on the use of a state-of-the-art CNN architecture to classify cervical images obtained with the hand-held Pocket colposcope, which can capture images of the same quality as an upright clinical colposcope [13, 31]. The dataset consists of 1,180 patient visits from a screened positive, referral population across 6 countries in North America, Central America, South America, Africa, and Asia. The backbone of the algorithm is a Resnet-18 architecture for automatic feature extraction and classification of high-grade disease. Features of the algorithm that were critical to performance included preprocessing to remove specular reflection and extraneous regions such as the speculum or vaginal walls, a class-balanced loss function to add weight to the positive class in order to improve sensitivity, and a multicontrast approach using both acetic acid and green light images. Using a combination of acetic acid and green light images, which accentuate acetowhitening and the vasculature, respectively, as input into the CNN algorithm resulted in an AUC of 0.87. The CNN algorithm paired with HPV testing will lead to increased specificity, fewer touch points to complete care, and reduced overtreatment, all of which are essential to achieving the WHO targets for cervical cancer prevention.

In comparison with the manuscripts listed in Table 1, this manuscript is unique in that explores deep learning classification using a dataset and device that are situated for clinical translatability, beyond those which are used in current cervical cancer detection literature. The training and test data is collected with an accessible device from multiple populations, and our use of a class-balanced loss preserves data heterogeneity while dealing with the real-world, low disease prevalence. Further, the incorporation of green light contrast, available with the Pocket and all standard colposcopes, to improve the AUC and sensitivity of the model comes at no additional cost to the patient or provider.

## 2. Results

We developed a baseline classification algorithm by identifying a method to remove clinically irrelevant features from the colposcopy images, establishing which CNN architecture should be used, and addressing class-imbalance within our dataset. We further improved the area under the curve (AUC) of our receiver-operator characteristic (ROC) curves and sensitivity of the model by including image pairs obtained with green light contrast.

**Figure 1:**
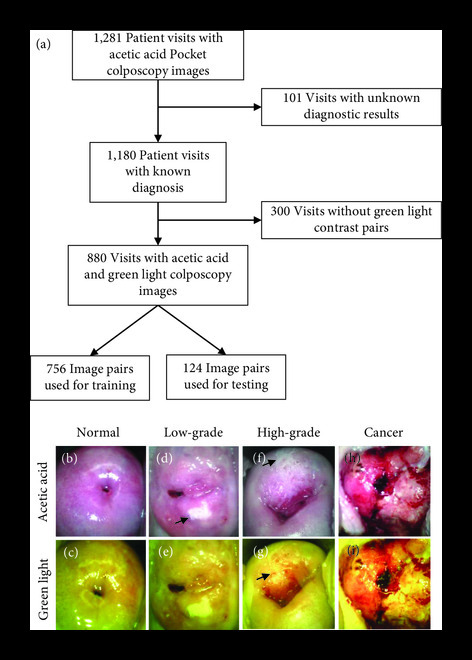
Dataset summary. The Pocket colposcopy dataset contained acetic acid images from 1,281 unique patient visits. Of those, 101 were excluded due to the absence of completed diagnostic results—either an indication of negative upon visual inspection or a biopsy or LEEP result. In total, 880 visits contained acetic acid and green light colposcopy pairs with their associated diagnostic result, which were used to train and test our model (a). Representative Pocket colposcope images using acetic acid and green light contrasts on cervixes diagnosed as normal under visual inspection with a colposcope from a screened Pap+ population (b, c), biopsy-confirmed low-grade precancer from a screened Pap+ population (d, e), biopsy-confirmed high-grade precancer from a screened Pap+ population (f, g), and biopsy-confirmed cancer from a screened VIA+ population (h, i).

The process used to determine the final number of patients included in our analysis is shown in Figure 1(a). The Pocket colposcope image database consists of images from 1,281 patient visits, with 1,180 visits resulting in a biopsy result or an indication of a colposcopically negative result. Of those, 880 visits had an acetic acid and green light pair for our analysis. Representative acetic acid and green light Pocket colposcopy image pairs are shown in Figures 1(b)–1(i). The normal cervix shows no acetowhite areas (Figure 1(b)), and the green light image shows little additional anatomical information—only the light pink of the underlying stroma (Figure 1(c)). Upon the development of a low-grade lesion, acetowhite areas develop after the application of acetic acid, such as that seen at five o’clock (Figures 1(d) and 1(e)). As the precancer progresses into high-grade, acetowhite areas remain and may grow, such as the lesion spreads across eleven to two o’'clock (Figure 1(f)), and abnormal, hairpin-like vascularization appears to support accelerated cell division (Figure 1(g) at eleven o’clock). The green light images accentuate the vasculature in addition to showing the acetowhite, as observed in Figures 1(e) and 1(g). In cervixes with cancer, there are morphological appearances as the original cervical epithelium is replaced with cancerous areas (Figures 1(h) and 1(i)). Note that before preprocessing, there are saturated areas of the image caused by specular reflection.

The 880 AA image-pairs summarized in Table 2 were split into a training set with 756 images and a test set with 124 images with a 24% and 16% positive sample rate, respectively. Images were randomized to the training and test sets; however, images from patients with multiple visits were allocated to the same split. A detailed diagnostic breakdown of the training and test data are given in Tables S1 and S2 of the Supplementary Materials.

**Table 2:** Pocket colposcopy dataset. The dataset is taken from a screened positive population across six different countries in Asia, Africa, the U.S., and South and Central America. Diagnostic breakdown varies by screening method (Pap-positive, visual inspection positive, and HPV positive), and there is an overall positivity rate of 23.1%.

	Screened Pap+ N=412 (%)	Screened VIA+ N=72 (%)	Screened HPV+ N=386 (%)	All screening methods N=880 (%)
Negative colposcopy impression (no biopsy)	12 (2.9)	8 (11.1)	76 (19.2)	**96 (10.9)**
Biopsy result				
Normal	211 (51.2)	7 (9.7)	167 (42.2)	**385 (43.8)**
Abnormal (benign/CIN1)	110 (26.7)	9 (12.5)	77 (19.4)	**196 (22.3)**
High-grade (CIN2/CIN3)	68 (16.5)	36 (50.0)	74 (18.7)	**178 (20.2)**
Cancer	11 (2.7)	12 (16.7)	2 (0.5)	**25 (2.8)**

Negative (<CIN2)	333 (80.8)	24 (33.3)	320 (80.8)	**677 (76.9)**
Positive (≥CIN2)	79 (19.2)	48 (66.7)	76 (19.2)	**203 (23.1)**

**Figure 2:**
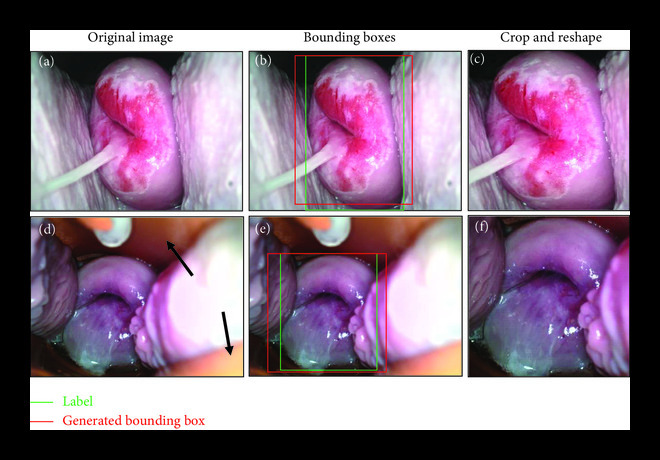
Representative YOLOv3 cervix detection results show removal of extraneous content from vaginal walls and speculum. YOLOv3 is used for automated cervix detection after training on images with a hand-drawn bounding box surrounding the cervix area. After automatic bounding box detection, the cervix is cropped and reshaped for entry into the classification network. Automated detection is necessary for the removal of extraneous content from vaginal walls (a–c) and the speculum pointed out with the black arrows (d–f). Both representative images are diagnosed as high-grade precancer and were captured from a Pap+ screened population.

The modified YOLOv3 cervix detection model resulted in an average precision of 0.997 on a test set of 83 acetic acid images. The automated detection model was used for the removal of the speculum and vaginal walls throughout all images prior to the training and test procedures. Representative examples of this preprocessing step are shown in Figure 2. The original 5MP image (a) is passed through the trained YOLOv3 cervix detector, which generates its own bounding box (b). For all images, both white light and green light, the YOLOv3-generated bounding box was used to crop the image. The cropped image was resized to 256×256×3 pixels before undergoing the specular reflection removal process. The process of removing extraneous content from both the vaginal walls and the speculum in cervical images is shown in Figures 2(d)–2(f).

**Figure 3:**
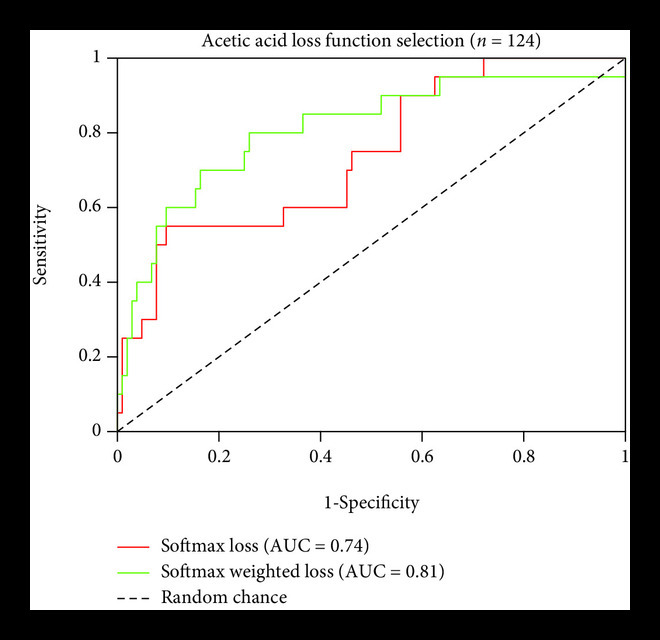
Loss function selection. Comparisons of test set performance metrics of the Resnet-18 model without and with the weighted loss function, showing an improved sensitivity and less variation when the model was trained on 756 AA images and tested on 124 AA images. Sensitivity and specificity values, were calculated using a CNN output threshold of 0.5.

The ResNet-18 CNN architecture with a cross-entropy loss function outperformed Inception-v3 and Vgg-16 (data not shown). The receiver operator curve (ROC) resulting from ResNet-18 had an area under the curve (AUC) of 0.74. Using a CNN output cutoff threshold of >0.50 to classify a positive image, the sensitivity and specificity were 0.30 and 0.95, respectively. To further improve the performance and address the severe class imbalance without the need for under sampling from the negative class, the original cross-entropy loss function was replaced with a label-weighted cross entropy loss function proposed by Cui et al. [32]. As opposed to traditional class balanced weights, which use an inverse or square inverse of the class frequency, Cui et al. use the effective number of samples to penalize the less frequent class. Implementing the weighted loss function improved the AUC from 0.74 to 0.81. The sensitivity remained at 0.30, and the specificity improved to 0.97. The metrics of Resnet-18 model with and without the weighted loss based on the effective number of samples are detailed in Figure 3.

Lugol’s iodine is not routinely used as a contrast agent and not readily available in most clinics where cervical cancer screening is performed; therefore, we removed Lugol’s iodine images from further analyses. The same training and test set split described in the first experiment was also used here; 880 acetic acid and green light image pairs were randomly divided into 756 image-pairs for the training set and 124 image-pairs for the test set such that the number of cases and control were approximately the same in both. The training set consisted of approximately 8% VIA+, 39% Pap+, and 52% HPV+ screened populations. The test set consisted of 8% VIA+ and 92% Pap+ screened populations. It should be noted that the images used in this testing set were the same as that used for testing the acetic acid-only images shown in Figure 3.

**Figure 4:**
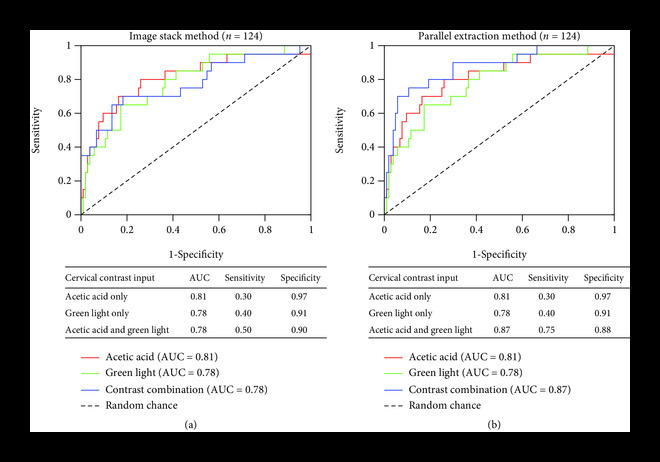
The combination of acetic acid and green light images improves overall classification performance compared to a single source of contrast for the parallel combination method. When training the network on image stacks of both acetic acid and green light images, the AUC does not show an improvement compared to acetic acid or green light alone (a). When features for both acetic acid and green light are extracted in parallel and then combined before the network’s fully connected layer, however, the AUC improves to 0.87 with the combination of green light and acetic acid images (b). In addition, there is a large performance improvement in sensitivity, increasing from 0.30 with acetic acid only to 0.75 when the combination of two contrast sources is used.

Two methodologies were explored for combining green light and acetic acid image pairs for classification: (1) a single input of a white and green light image stack and (2) separate inputs of green and white light images for parallel feature extraction. As shown in Figure 4(a), the image stack method resulted in an ROC with an AUC of 0.78, failing to outperform the ROCs using either acetic acid (0.81 AUC) or green light images only (0.78 AUC). The combination of acetic acid and green light images improved the sensitivity to 0.50 with a minor decrease in specificity to 0.90. Combining acetic acid and green light images using parallel feature extraction resulted in the highest AUC of 0.87 and outperformed the ROC AUCs of both the acetic acid images (0.81 AUC) and green light images (0.78 AUC) as shown in Figure 4(b). Similar to the image stack method, the parallel input method resulted in a decrease in specificity from 0.97 to 0.88 and 0.91 to 0.88 for acetic acid or green light images, respectively. The improvement in AUC and sensitivity with the parallel extraction method indicates that use of the contrast image pairs results in a model capable of learning on class-imbalanced datasets from a screen-positive population.

Further, we compared our final method using the parallel feature extraction technique with a more commonly used technique to weight the loss function—the inverse class balance. We found that simply weighting by the inverse class balance did not perform as well as using the effective number of samples, achieving an AUC, sensitivity, and specificity of 0.83, 0.40, and 0.92, respectively.

## 3. Discussion

We adapted convolutional neural networks (CNN) for automated detection of high-grade precancerous or cancerous cervixes using images collected via an FDA-cleared, low-cost, and portable Pocket colposcope. Both acetic acid contrast (with white light) and the vascular features (using green light) were captured with the Pocket colposcope, enabling multi-contrast based image diagnosis. Without training and testing on identical cervical cancer datasets, it is challenging to make direct comparisons across approaches. Instead, many studies, including the one presented here, use their internal dataset to compare the marginal improvements of each additional method proposed. In our case, the use of a class-balanced loss and the incorporation of green light improve the model AUC from 0.74 to 0.87. The best AUC falls in the upper range of what is reported in current literature (Table 1).

Thus far, publications that report on the use of CNNs for the classification of colposcopy images are trained and tested on digitized cervigram film images or those digitally captured with a conventional colposcope (Table 1). This study is distinct in that the algorithm is developed on images with comparable quality to those obtained with a conventional colposcope but captured using an accessible device; the Pocket colposcope has a 2- to 3-fold higher Weber’s contrast ratio than a standard colposcope model, 27.8 *μ*m resolvable feature size, and 30.7 mm diagonal field of view. The images and the corresponding histopathological ground-truths that were used to train and test our approach come from 880 patient visits across 1,760 paired acetic-acid and green-light images. Patients were enrolled from a screen-positive population—therefore, the cervixes in the dataset were either confirmed as visually (VIA+), morphologically (Pap+), or molecularly (HPV+) abnormal, and the diagnosis rate for high-grade precancers and cancers combined was 23% (406 paired images).

While specular removal of Pocket colposcopy images has been reported previously [33], we show here the utility of the YOLOv3 automated cervical detection algorithm as a preprocessing step to further reduce clinically irrelevant features from the surrounding area prior to cervical cancer classification. The larger cervix-to-image ratio allows for the elimination of the YOLOv3 small- and medium-scale detection arms, which reduces the size of the detection network.

The prevalence of precancers in the general screening population is low; for every 100 people screened from the general population, approximately 10-20% are screen positive [1, 27–29]. We tackled this problem of class imbalance by incorporating a weighted loss based on the effective number of samples, which improved the AUC and substantially boosted the sensitivity of the CNN algorithm. Lastly, to further improve sensitivity, we paired the acetic acid images with their corresponding green-light images. Previous studies analyzing provider performance in image-based diagnosis have shown an improvement in classification accuracy when they were provided additional sources of contrast [14, 34].

We performed two methods of incorporating the acetic acid and green light contrast-pairs: first, by concatenating the two RGB images into a 6-image stack, and second, by introducing them into the network separately to be reduced to feature maps before concatenation followed by classification. The latter method increased the AUC from 0.81 for acetic acid alone to 0.87 for the contrast combination, and it improved the sensitivity from 0.30 to 0.75. There are several reasons for the performance improvement with the parallel feature extraction method compared to the image-stack method. The primary issue with image stacks is that important spatial information may not align between the two images; even if one were to achieve perfect image registration, acetowhite regions (highlighted in the acetic acid image) and abnormal vasculature (highlighted in the green light image) could appear in separate locations on the cervix, leading to a conflict in the algorithm’s spatial attention and thereby lead to a compromise within both sets of features. This issue is avoided with the parallel feature extraction method, as weights can be tuned individually. Given the increase in the number of tunable parameters, the parallel extraction method can potentially overfit the training data. To avoid this issue, we performed our analysis on the same test set of 124 images to allow us to select the most generalizable model.

When translating the ROC results into a single sensitivity and specificity value, multiple thresholds could be selected. While existing literature is not always clear on which cutoff is used, the default threshold is 0.5, giving equal weight to the image being negative or positive. In practice, this threshold could be changed to favor either improved sensitivity or specificity; however, for consistency, we used the 0.5 cutoff in all our experiments. For further exploration of the improvements provided by our best performing model, we evaluated the sensitivity and specificity values obtained using the Youden’s index criteria, which represents the top-left most point on the ROC curve. The max index and corresponding sensitivity and specificity values were 0.63, 0.75, and 0.88, respectively (note that the results from the Youden index happen to align with the 0.5 cutoff shown in Figure 4(b)).

It is important to note that given the low prevalence of disease in our training and test sets, it was natural for the algorithm to favor higher specificity at the cost of sensitivity. Therefore, our efforts focused on improving the overall AUC in order to improve the sensitivity at minimal costs to the specificity. In addition, given that our target population are patients that may be in areas that are harder to access frequent screening, a high sensitivity would be more important than if screening were able to occur more regularly, as is the case with the Pap smear in the U.S.

Our findings are correlated with previous work in which a machine-learning algorithm was developed using multicontrast images with the Pocket colposcope, The algorithm was trained and tested on a small set of the Pocket colposcope dataset (128 patients) [33] using cross-validation. The cervix image was manually cropped to remove clinically irrelevant features from the speculum and vaginal walls, and four gray-level cooccurrence matrix (GLCM) features were extracted: homogeneity, energy, contrast, and correlation. Sixty-five color summary statistics were extracted after Gabor segmentation creating a total of 69 features. For each contrast source, a forward sequential feature selection algorithm performed 5-fold cross-validation to minimize classification error as individual features were added sequentially. Selected features were used to in a logistic regression classifier, and the model was validated using five-fold cross validation with biopsy results as the ground truth. Similar to the findings of our current study, we demonstrated that a combination of acetic acid and green light images outperformed either contrast alone for the classification of high-grade precancerous lesions. The combination improved the ROC AUC to 0.86, up from 0.76 (for acetic acid alone) or 0.81 (for green light alone) on a cross validation dataset of 128 patients.

Other studies have incorporated a multicontrast approach to improve deep learning performance. Most notably, Yu et al. used a colposcopy dataset from 679 patients, totaling 282 instances of normal cases, 129 cases of low-grade lesions, 196 cases of high-grade lesions, and 72 cases of cervical cancer [25]. Each patient had a time series of acetic acid images obtained every 30 seconds across three minutes, a single green filter image, and a single Lugol’s iodine image. The deep learning model processed each contrast source through a different CNN to extract three feature vectors, which were then combined into a fully connected layer prior to the softmax layer. Using a test set of 20% of their data, they showed the highest performance using their combination model (0.90 AUC) compared to classification based solely on an individual contrast source. It should be noted however that the colposcopy image dataset in their study was from a single-patient population, all screened in the same manner, whereas our dataset provided addition classification challenges given the wide range of prescreening practices employed based on the availability of laboratories and testing across the different countries and populations.

Our study used a database that is true to real-world scenarios—that is, prescreened individuals imaged with an accessible colposcopy device. Complicated examples that are common in this subset, such as cervixes with low-grade lesions or cervicitis, were not excluded from training and testing. In this triaged population, compared to the general population, it may be more common for images to be histopathologically negative for precancer and yet appear to be inflamed or in a visually “grey zone.” In this case, we expect more overlap between the feature spaces between negative and positive images—in other words, the histology confirmed negative may have features of abnormality given that this was performed on screen-positive population. Another potential complication is that negative cervices could appear abnormal owing to normal changes in physiology corresponding to patient age. For example, conditions such as ectropion (characterized by a large area of red columnar tissue) could play a role in misdiagnosis—the highly texturized and red columnar tissue could appear similar to the high grade precancerous or cancerous cervix. Another benign condition, cervicitis, similarly creates inflammation and more texturization. Indeed, one-third of the labeled negative cervixes that were misclassified in our final model were diagnosed as cervicitis. To create an algorithm that is more robust, future studies may consider either a balance of these benign conditions among training and test images, a multi-class classification approach, or consider age as an additional variable as ectropion is more common in younger patients. Operation-wise, it would be beneficial to incorporate additional preprocessing and decision-making steps, such as narrowing down to a region of interest. This can be achieved through segmentation prior to training for classification.

The use of ImageNet dataset, consisting of everyday objects and animals, to pretune the weights of the Resnet-18 model does teach the model to pick up characteristics common to all images, such as edges and shapes. However, the classes present in the ImageNet dataset are still drastically different than the positive and negative classes of colposcopy images, and the model therefore has a large knowledge gap to fill with only roughly 1,000 image examples. In the future, this can be improved by pretraining the network on a dataset that shares similar features to our Pocket colposcopy images. This could be cervical images made public through a Kaggle competition and accompanied by labels describing the cervix’s transformation zone [35] or from standard colposcopy databases. In addition, an unlabeled approach, such as an autoencoder, could be explored in which the network learns to reduce images down to their most important features, and those weights could then be used as a starting point for the classification task.

In summary, we present a deep learning approach to automate diagnosis of high-grade precancer or cancer, using colposcopy images captured from a screened-positive population. We optimized our model to handle class-imbalance using a class-balanced loss function and the addition of acetic acid and green light cervical contrast images to achieve an area under the receiver-operator characteristic curve of 0.87, and a sensitivity and specificity of 75% and 98%, respectively. This study is the first to use such a large dataset, consisting of 203 CIN2+ cases, imaged with a portable, FDA-cleared, and low-cost device, thereby building off the work that has been performed on the Guanacaste dataset, which uses scanned cervigram images [21]. Even though our overall sample size is small, the number of CIN2+ samples from this dataset is comparable to the number in the Guanacaste data. Despite additional complexities in our training and test data compared to the Guanacaste dataset, we achieved a similar sensitivity and specificity on a screen-positive population. In the case where self-HPV testing is the primary screening tool, an HPV-positive follow-up with automated visual diagnostics via accessible Pocket colposcopy could streamline the overall process and reduce the number of clinic visits and gynecologic examinations.

## 4. Materials and Methods

### 4.1. Pocket Colposcopy Image Dataset

The dataset consists of cervix images captured by clinicians with the mobile Pocket colposcope, described in detail in Lam et al. [31]. In summary, the handheld device consists of a CMOS camera surrounded by a ring of white and green LEDs. During use, a clinician inserts the Pocket colposcope through the speculum, uses a sliding control to adjust the optical zoom, and then, captures a 5MP RGB image with either white light (to image the cervix stained with acetic acid) or green light (to highlight the vasculature).

In total, Pocket colposcopy images were pooled from clinical sites across six countries to create the dataset of 1,180 unique patient visits with which to develop our deep learning model. Acetic acid and green light colposcopy pairs were imaged from 880 patients. All images were obtained from a referral population of either persons positive upon initial visual inspection of the cervix with acetic acid (VIA+), positive for an abnormal pap test result (Pap+), or positive for high-risk HPV (hrHPV+). If multiple images were taken for a given source of contrast, the image with the most cervix visibility and focus was selected for use.

Pathology labels were retrospectively obtained from previous clinical studies from all patients with a visually appearing abnormal area [14, 36]. Standard of care clinical diagnoses were made from images obtained with either a standard clinical colposcope or a digital camera and not with the investigational device. After imaging, the healthcare provider obtained a biopsy of up to one to three suspicious areas. The biopsy samples were submitted to a local pathology lab for histopathological analysis, and the samples were diagnosed as either normal or benign (including cervicitis or condyloma); low-grade precancer (including cervical intraepithelial neoplasia grade 1, CIN1); high-grade precancer (including CIN2 or CIN3); or cancer (including adenocarcinoma or squamous cell carcinoma). For patients who had no apparent lesion at the time of visual inspection, the clinician did not perform a cervical biopsy, and the image was classified as colposcopically or visually negative. For the purposes of pathologic diagnosis, the most severe diagnosis within a patient (from whom more than one biopsy was obtained) was used as the gold standard label. Both cervical images from which no biopsy was obtained (owing to the absence of lesions), and those that were deemed negative after pathologic diagnosis of the biopsy were labeled as normal for the purposes of training the algorithm. All patients included in the study signed an informed consent form. Images were collected under Duke University Medical IRB approved protocols (Pro00008173 and Pro00052865), or IRB approvals were obtained from PATH’s Research Ethics Committee and the Comité de Ética en Investigación Biomédica (CEIB) of Universidad Nacional Autónoma de Honduras in Tegucigalpa. The studies are registered on ClinicalTrials.gov (NCT02477124, NCT00900575, and NCT03510273).

### 4.2. Summary of Model

**Figure 5:**
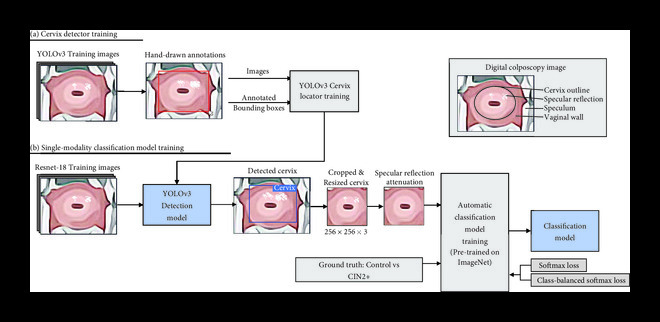
Key steps of the analysis process. Initially, hand-drawn bounding boxes were used to label cervix locations to train a YOLOv3 cervix detector to automatically remove extraneous content from the colposcopy image (a). Next, the trained cervix detector automatically generated the bounding boxes for the training images of the classification algorithm. The bounding boxes were used to crop and resize the cervix images preceding the removal of specular reflection. The preprocessed image and the associated label were then used to train the classification model, which was tested with and without a weighted loss function (b).

An outline of the steps of the algorithm is shown in Figure 5. To remove the speculum and vaginal walls from the image, a subset of images in the Pocket colposcopy dataset were used to train and validate a cervix detector algorithm with a YOLOv3 CNN backbone (Figure 5(a)) [37]. The images were then passed through the trained YOLOv3 cervix detector to obtain cervix bounding box coordinates, which were used to crop the cervix and resize the image (Figure 5(b)). Prior to training the classification algorithm, spots of specular reflection, caused by light shining on the moist surface of the cervix, were attenuated using a Laplacian infill technique [33, 38]. After preprocessing was complete, acetic acid images were used to train the classification model and select the highest-performing CNN architecture and loss function. The optimized methodology was used to train and test three different sets of data: white light images of acetic acid only, green light images only, and both. The dual-contrast images were evaluated in two different formats—as either stacked images or by having their features extracted in parallel before having their feature maps concatenated for classification.

### 4.3. Image Preprocessing

Cervix bounding box coordinates were automatically generated using the YOLOv3 [37] detection model. While the original model utilizes three multiscaled detection branches to capture small, medium, and larger image objects, the small- and medium-scale detection branches were removed due to the cervix taking up a large portion of the Pocket colposcopy images. Another important feature of YOLOv3 detection is the anchor box. First, anchor boxes of various, predefined sizes and ratios are placed on the image. Intersection over union (IoU) is the ratio of the area of intersection (area of overlap) and the area of union (the total area of the two boxes combined less the area of overlap) of the manually annotated box and the box generated by the YOLOv3 object detection model. If an anchor box achieves an IoU greater than 0.5, the algorithm is told to predict the object. Because successful training relies on achieving at least a 0.5 IoU, we modified the sizes of several of the anchor boxes to resemble larger, more square-shaped boxes to approximate the proportions of a cervix for greater training success.

The modified YOLOv3 model was trained on 800 acetic acid-stained cervix images with manually annotated bounding boxes positioned to exclude clinically irrelevant features from the speculum and surrounding vaginal walls. The fully trained model was validated with a separate set of 83 randomly selected images. True positives were defined as a prediction resulting in an IoU of greater than 0.5. The results were summarized in a precision-recall curve, where precision is the percentage of positive predictions that is truly positive, and recall is the percentage of true positives that were correctly classified as positive. Next, the precision (y-axis) vs. recall (x-axis) was plotted. The average precision, which was the AUC, was calculated. All training and test data was passed through the trained YOLOv3 model to automatically generate bounding box coordinates, which were then used to crop and resize the images to 256×256×3 RGB images for input into the CNN. For any cases where YOLO did not achieve a certainty of greater than 0.6 during image detection, the detection algorithm would center crop the image and resize the image by 256×256 to ensure capture of the cervix region. Therefore, before input into the classification algorithm, no cervix images were misdetected.

During colposcopy imaging, the cervix’s moist epithelium commonly results in areas of specular reflection that are best attenuated during image preprocessing. The specular attenuation process is described in detail by Das et al. [38]. In summary, binary masks were automatically created for each image using selected pixels from the red, green, and blue color channels that were all greater than 220 to delineate the regions of interests. The mask was used to locate the areas on the original image for specular removal, which were then filled using a Laplacian infill—performed here with MATLAB R2020a but has equivalents on publicly available programming languages.

### 4.4. Architecture Selection

We explored the use of CNNs to achieve a binary classification task of negative for high-grade precancer or diagnosed positive for high-grade precancer or cancer (CIN2+). Acetic acid images were used to select the CNN classification model because they represent the most frequently used and accessible method of colposcopy contrast [39]. Three high-performing CNN architectures were trained and tested: ResNet-18 [40], Vgg-16 [41], and Inception-v3 [42] with a cross-entropy loss. We adopted the weights pretrained on ImageNet [43], a large visual database designed for use in visual object recognition software research, and fine-tuned those weights on the acetic acid images.

Given the limited data available for our task (880 image pairs) and the low-prevalence of the positive class, we opted to use the majority for training (85%) and the remaining images as a validation-test set (as performed by Hu et al. and Yan et al.) [21, 26]. Specifically, we tuned model parameters on the same training set for each experiment and evaluated the tuned parameters on the test set during training. The parameters that were chosen for each experiment were those that were most generalizable to the test set (i.e., resulted in the highest test set AUC performance). Like the training set, the test set of 124 image pairs remained consistent across all experiments to achieve internally consistent results.

### 4.5. Loss Function Selection

Due to the class imbalance between positive and negative samples, the loss function was replaced with a weighted cross-entropy loss described by Cui et al. [32]. Often, reweighting the loss function is performed using the inverse class frequency; the weight would be equal to one over the number of samples in the class, normalized across the different classes. However, since there is information overlap between data, the marginal benefit model gain from data decreases. This implies that the weight (or imbalance ratio) cannot be fully characterized by the number of samples. Instead, we use a class-balanced loss function that is based on the *effective* number of samples, wherein the effective number represents the overlap or repetition in knowledge that a new instance of a given class provides. The formula for the effective number is given in Equation (1), in which $E_{n}$ is the effective number, the hyperparameter $\beta \in \left.\left[0,1\right.\right),$and n is the instance number in a class. The new weighted cross-entropy loss function is given in Equation (2), in which C is the total number of classes, z is the predicted output, and y is the class label.

(1)En=1−βn1−β,(2)CBz,y=−1−β1−βny∑j=1Cyjlogzj.

For our selected model, we went on to compare the loss function weighted based on the effective number of samples, with a more commonly used technique to weight the loss function—the inverse class balance.

### 4.6. Multicontrast Inputs

**Figure 6:**
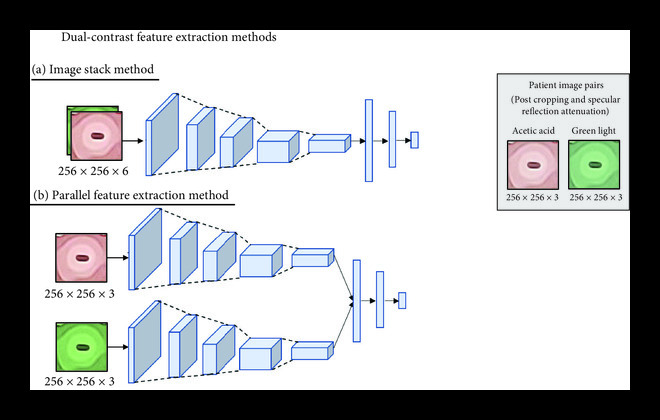
Dual-contrast feature extraction method. Using the Resnet-18 CNN architecture, we explored two approaches for combining acetic acid and green light colposcopy images taken from a single patient. The “image stack” method concatenates the two RGB images to create a 6-channel input into the neural network (a). The “parallel feature extraction method” tunes two networks, one for each contrast source, to extract features independently before combining the fully connected layers for classification (b).

To assess the increase in performance when a combination of images with different contrast sources were used, we conducted two tests: first, we trained the CNN model with image stacks of two RGB images (one of each contrast type) so that image pairs could be evaluated by the model simultaneously (Figure 6(a)). Specifically, we modified the first layer of the selected model (shown in Figure 6(b) under “Automatic Classification Model Training”) to a convolutional layer that had six input channels. Second, we tested a parallel processing method in which the acetic acid and green light images were input into two separate models for feature extraction before having their feature maps merged for the fully connected and final classification layers of the network (Figure 6(b)). To evaluate model improvements, absent from patient variation in test set images, we utilized the same test set of 124 images used during architecture selection for both approaches. For each method explored, we compared the results to the CNN model trained on each source of contrast individually (as performed in the acetic acid-only experiment).

### 4.7. Statistical Methods

Definitions for the evaluation indicators are listed in Table 3. The YOLOv3 cervical detection method is evaluated using the mean average precision, defined as the area under the precision-recall curve, $p\left(r\right)$, with the intersection over union threshold (α) of 0.5 or greater indicating a true positive. Classification performance metrics include the area under the receiver-operating characteristic curve (AUC) and the sensitivity and specificity. The CNN’s output, in the form of pseudoprobability scores, was converted to a binary result for each instance using a default cutoff of 0.5 for model selection and used to count the total number of true-positive, true-negative, false-positive, and false-negative instances and the corresponding sensitivity and specificity. In practice, the threshold could be changed to favor either improved sensitivity or specificity; therefore, we selected the best performing model based on the AUC results.

**Table 3:** Evaluation indicator definitions.

Evaluation	Definition
Mean average precision	$\int_{0}^{1}p\left(r\right)dr$, α=0.5
Sensitivity	$\text{True}\;\text{positives}/\left(\text{true}\;\text{positives}+\text{true}\;\text{negatives}\right)$
Specificity	$\text{True}\;\text{negatives}/\left(\text{true}\;\text{negatives}+\text{false}\;\text{positives}\right)$

## Data Availability

All relevant data that support the findings of this study are within the paper and its Supporting Information files. The patient images and pathology labels used in this study are restricted by the Institutional Review Board in order to protect patient privacy.

## Supplementary Materials

Supplementary Materials Table S1: Pocket colposcopy training dataset. Table S2: Pocket colposcopy testing dataset.
