# Supplementary material for: Multicontrast Pocket Colposcopy Cervical Cancer Diagnostic Algorithm for Referral Populations
Source: BME Front. 2022 Aug 25;2022:9823184. doi: 10.34133/2022/9823184 (PMC10521679; doi:10.34133/2022/9823184)
Supplement: Supplementary Materials — Table S1: Pocket colposcopy training dataset. Table S2: Pocket colposcopy testing dataset. [file 9823184.f1.pptx]

## Slide 1
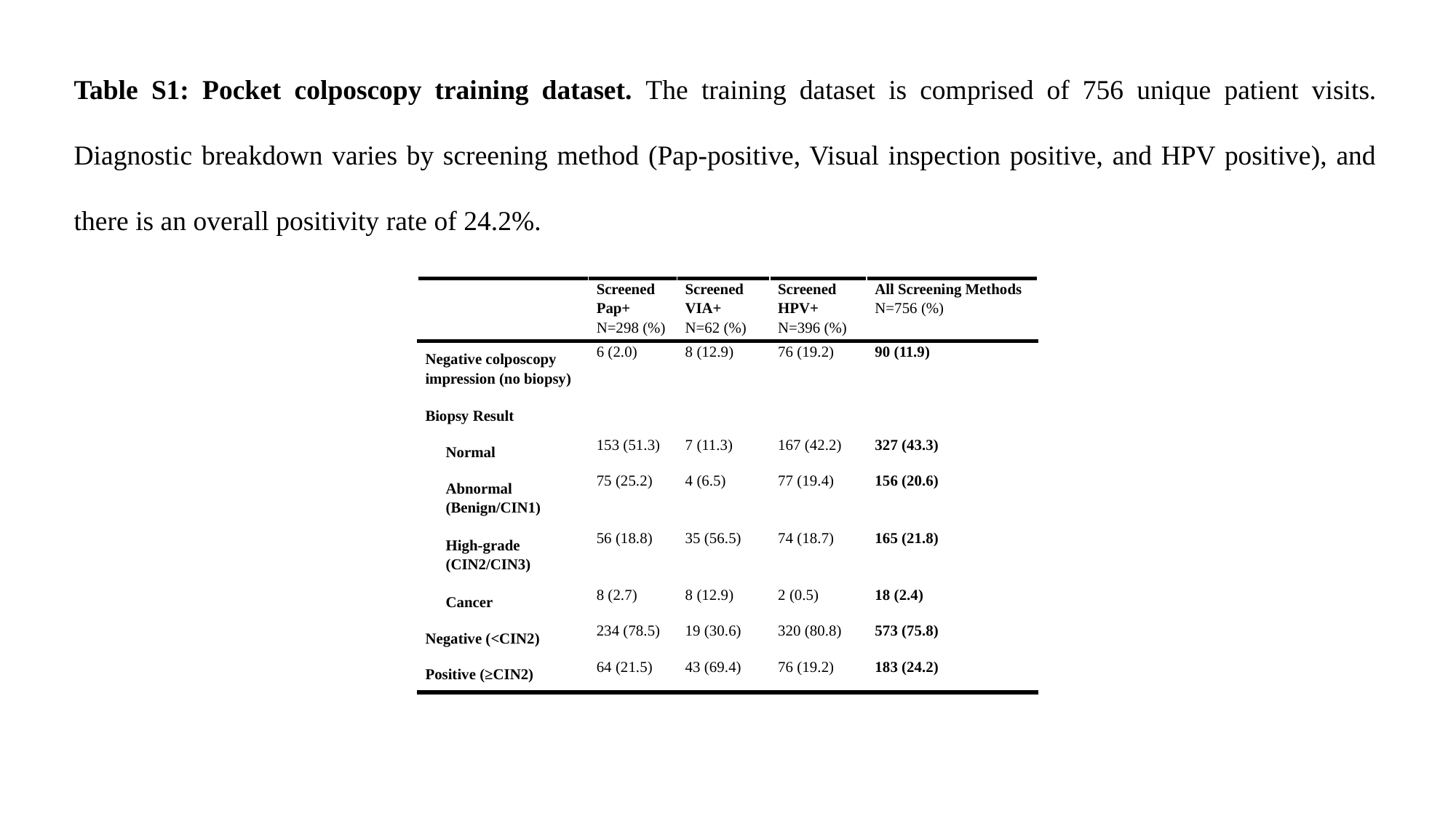

Table S1: Pocket colposcopy training dataset. The training dataset is comprised of 756 unique patient visits. Diagnostic breakdown varies by screening method (Pap-positive, Visual inspection positive, and HPV positive), and there is an overall positivity rate of 24.2%.
| | Screened Pap+ N=298 (%) | Screened VIA+ N=62 (%) | Screened HPV+ N=396 (%) | All Screening Methods N=756 (%) |
| --- | --- | --- | --- | --- |
| Negative colposcopy impression (no biopsy) | 6 (2.0) | 8 (12.9) | 76 (19.2) | 90 (11.9) |
| Biopsy Result | | | | |
| Normal | 153 (51.3) | 7 (11.3) | 167 (42.2) | 327 (43.3) |
| Abnormal (Benign/CIN1) | 75 (25.2) | 4 (6.5) | 77 (19.4) | 156 (20.6) |
| High-grade (CIN2/CIN3) | 56 (18.8) | 35 (56.5) | 74 (18.7) | 165 (21.8) |
| Cancer | 8 (2.7) | 8 (12.9) | 2 (0.5) | 18 (2.4) |
| Negative (<CIN2) | 234 (78.5) | 19 (30.6) | 320 (80.8) | 573 (75.8) |
| Positive (≥CIN2) | 64 (21.5) | 43 (69.4) | 76 (19.2) | 183 (24.2) |

## Slide 2
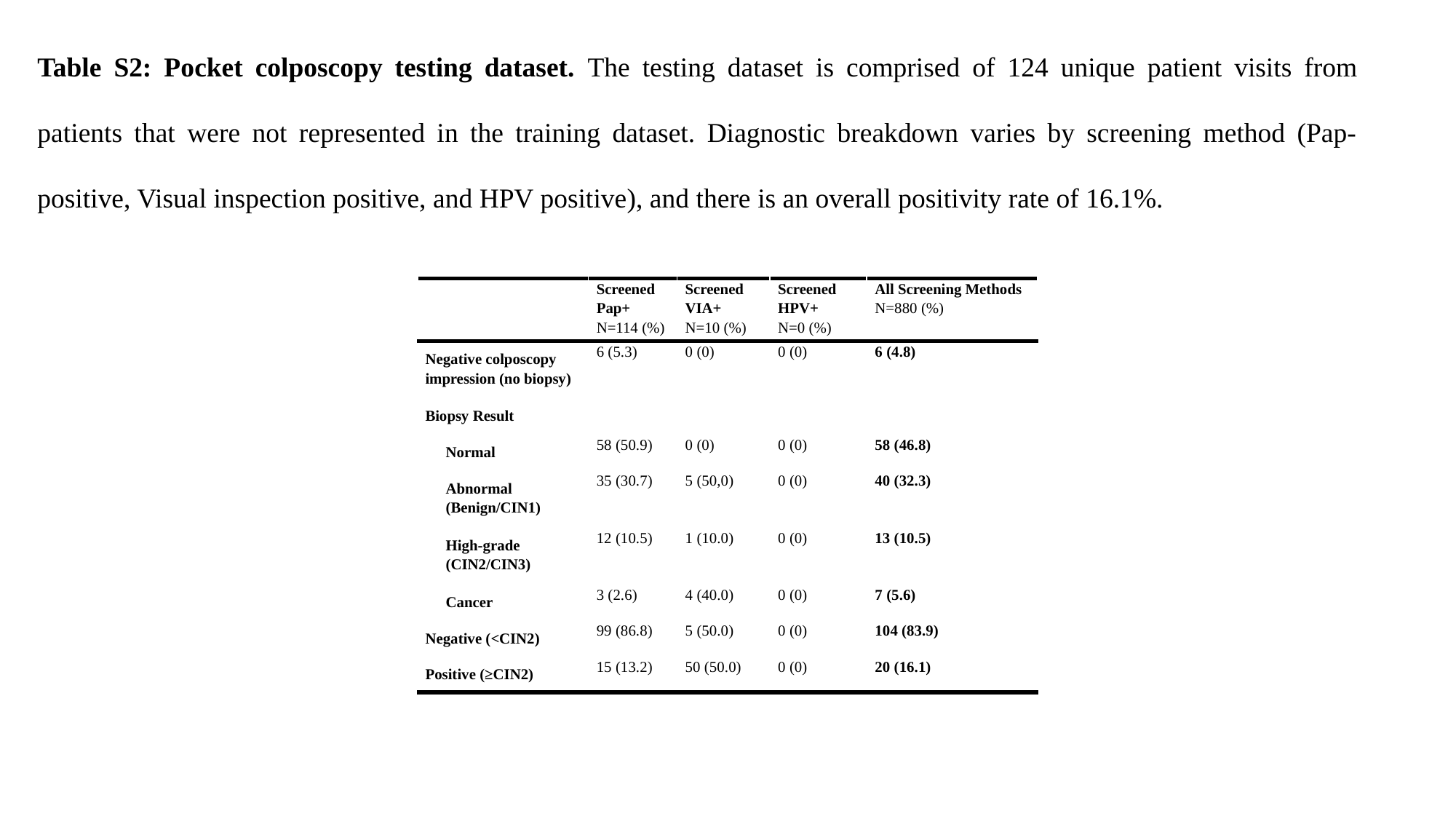

Table S2: Pocket colposcopy testing dataset. The testing dataset is comprised of 124 unique patient visits from patients that were not represented in the training dataset. Diagnostic breakdown varies by screening method (Pap-positive, Visual inspection positive, and HPV positive), and there is an overall positivity rate of 16.1%.
| | Screened Pap+ N=114 (%) | Screened VIA+ N=10 (%) | Screened HPV+ N=0 (%) | All Screening Methods N=880 (%) |
| --- | --- | --- | --- | --- |
| Negative colposcopy impression (no biopsy) | 6 (5.3) | 0 (0) | 0 (0) | 6 (4.8) |
| Biopsy Result | | | | |
| Normal | 58 (50.9) | 0 (0) | 0 (0) | 58 (46.8) |
| Abnormal (Benign/CIN1) | 35 (30.7) | 5 (50,0) | 0 (0) | 40 (32.3) |
| High-grade (CIN2/CIN3) | 12 (10.5) | 1 (10.0) | 0 (0) | 13 (10.5) |
| Cancer | 3 (2.6) | 4 (40.0) | 0 (0) | 7 (5.6) |
| Negative (<CIN2) | 99 (86.8) | 5 (50.0) | 0 (0) | 104 (83.9) |
| Positive (≥CIN2) | 15 (13.2) | 50 (50.0) | 0 (0) | 20 (16.1) |
